# Psychometric assessment and behavioral experiments using a free virtual reality platform and computational science

**DOI:** 10.1186/s12911-016-0276-5

**Published:** 2016-03-19

**Authors:** Pietro Cipresso, Silvia Serino, Giuseppe Riva

**Affiliations:** 1 Istituto Auxologico Italiano, Applied Technology for Neuro-Psychology Lab, Milan, MI Italy; 2 Department of Psychology, Università Cattolica del Sacro Cuore, Milan, Italy

**Keywords:** Virtual reality, Psychometrics, Behavioral measurement, Tools, Psychophysiology

## Abstract

**Background:**

Virtual Reality has been extensively used in a wide range of psychological experiments. In this study, we aimed to introduce NeuroVirtual 3D, a platform that clinicians could use free of charge.

**Implementation:**

The platform we developed relies on NeuroVR software, but we extended it to apply to experiments. The software is available free of charge to researchers and clinical practitioners who can also use a large number of virtual environments and objects already developed.

**Results:**

The platform has been developed to connect to virtually every device ever produced by the means of Virtual-Reality Peripheral Network (VRPN) protocols; however, a number of these have already been included and tested in the platform. Among the available devices, the Microsoft Kinect low-cost sensor has already been configured for navigation through the virtual environments and to trigger specific action (sounds, videos, images, and the like) when a specific gesture is recognized, e.g., a step forward or an arm up. A task for neglect and a task for spatial abilities assessment were already implemented within the platform. Moreover, NeuroVirtual 3D integrated a TCP-IP-based module (bridge) to collect the data from virtually any existent biosensor (Thought-Technology, Zephyr and StarStim devices have already been included in the platform). It is able to record any psychophysiological signal during any experiment using also the computed indices in real time.

**Conclusions:**

NeuroVirtual 3D is able to record external and internal (e.g., coordinates, keys-press, timestamp) data with a millisecond precision, representing *de facto* the most advanced technology for experimental psychology using virtual environments available without the needs to program code.

## Background

Virtual reality has dramatically expanded in the last decade [5, 21, 22, 30]. Furthermore, in the last few years, several important innovations emerged in terms of software and hardware in the computer gaming field. Interestingly, many gaming devices have found clinical uses, as clinicians and engineers collaboratively adapt gaming to clinical protocols [6]. An example is Microsoft Kinect, a device widely used in clinical applications, such as post-stroke rehabilitation and cognitive tasks driven by gestures [15, 32]. Unfortunately, the biggest problem with such an approach is the complexity of creating efficient clinical protocols and experimental design without the help of engineers [7, 8, 10].

Unlike years ago, the main problem with virtual reality today is not the cost of the hardware but that of the software. The equipment, consisting of a computer with adequate graphic card, a head-mounted display (HMD), and a Kinect, can be purchased for less than 1,500 USD. However, the cost of programming a personalized protocol or creating an experimental design involves a team of engineers and user specialists cooperating with a psychologist to design new technologies, which means spending tens of thousands of dollars per experiment [21, 22, 25, 34].

In particular, the main problem with using virtual reality (VR) and new devices is customization [1, 23]. Researchers interested in using VR need flexibility. The more flexibility they need, the greater the complexity required, which in turns requires advanced programming skills, computer graphic expertise, user design specialization, usability and ergonomic issues, software developer kit (sdk) integrators, engineering, 3D architectural knowledge, and so on.

Alternative low-cost solutions are also available, but only without flexibility. This means that it is possible to use virtual environments and devices as long as they are made by the gaming industry, which is the approach most commonly used in serious game environment [7, 10, 33]. In this study, a clinical protocol or experimental procedure is adapted to the existing products. However, researchers, and particularly clinicians, have always encountered problems in adapting procedures and protocols, which by definition follow strict processes that can rarely be modified.

Regarding gaming, social virtual environments (e.g., Second Life) also face flexibility problems that require software skills in order to program protocols and procedures (even when the 3D graphical manipulation is made simple by “drag-and-drop”). Increased number of social virtual environments are generally open to the public and thus are susceptible to attacks on personal data, so their use is not suggested for clinical or experimental settings, where users’ privacy must be strictly guaranteed [12, 30].

## Implementation

Given the difficulties that researchers, and particularly clinicians, who lack an engineering background face, we aimed to create a platform designed by clinicians for clinicians. The main idea was to provide researchers with two distinct modules:

1. An editor module to create, edit, manipulate and update environments, 3D objects, images, sounds, video, environments, lighting, and all connected devices; and
2. A player module to administer protocols to the research participants by using a PC monitor, HMD, or other device, and interact with the external devices configured in that protocol.

Within the editor module, researchers can build protocols in a very simple way. Using a drag-and-drop procedure, they can import, move, rotate, and scale all kinds of 2D and 3D objects, images, and videos, triggering them for time- or space-specific events.

In the following section, we describe the main characteristics of the resulting NeuroVirtual 3D platform, which is available for free with tens of virtual environments and hundreds of objects and without any limitation in time or functioning, allowing an infinite range of experimental designs and clinical protocols for basic and applied psychological research.

## Results and discussion

The newly developed platform expanded the features of the software NeuroVr 2.0 (http://www.neurovr.org/neurovr2/) [20, 22, 24–26], a previous version, which allowed us to test experimentally in clinical settings the potentiality of VR and the emerging needs, which have been integrated systematically in the last years.

NeuroVirtual 3D platform is an open-source based software, providing researchers with a cost-free virtual environment builder to allow non-expert users to easily create a virtual scene that would best suit the needs of the experimental design and clinical protocols.

**Fig. 1:**
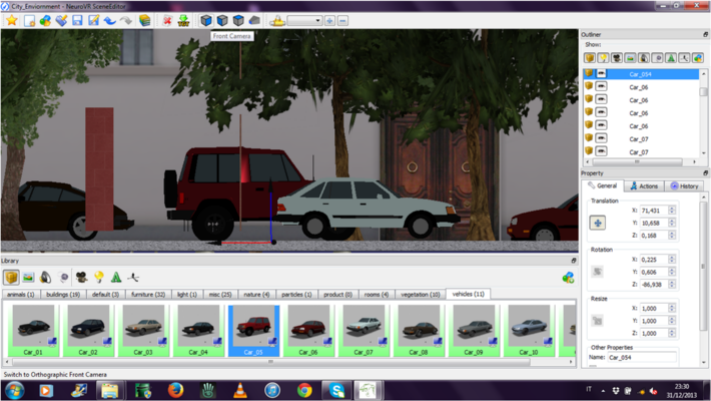
Drag and drop of a 3D object (a car) in the virtual environment in front camera view. Once imported in the scene, the object can be moved, rotated, and resized along the three axes, using the mouse (blue, red, and yellow line at the center of the object) or using values (on the bottom right side of the screenshot)

The editor and player modules are implemented using open-source components that provide advanced features, including an interactive rendering system based on OpenGL, which allows for high quality images. NeuroVirtual Editor is realized using QT libraries, a cross-platform application development framework used widely for the development of GUI programs. Using the editor, researchers can chose from a rich database of 2D and 3D objects and place them easily into the pre-designed virtual scenario by using an icon-based interface (no programming skills are required) (Fig. 1). Moreover, editor allows overlying the 3D scene video with a transparent alpha channel.

Thanks to these features, clinicians and researchers have the freedom to run, copy, distribute, study, change, and improve the NeuroVR contents to benefit the entire VR community. The other component of NeuroVR is the player, which allows navigating and interacting with the created Virtual Environments (VEs) using the NeuroVR editor. When running a simulation, the system offers a set of standard features that can increase the realism of the simulated scene. These include collision detection to control movements in the environment, realistic walk-style motion, advanced lighting techniques for enhanced image quality, and streaming of video textures using alpha channel for transparency. The player can be configured for two basic visualization modalities, immersive and non-immersive.

**Fig. 2:**
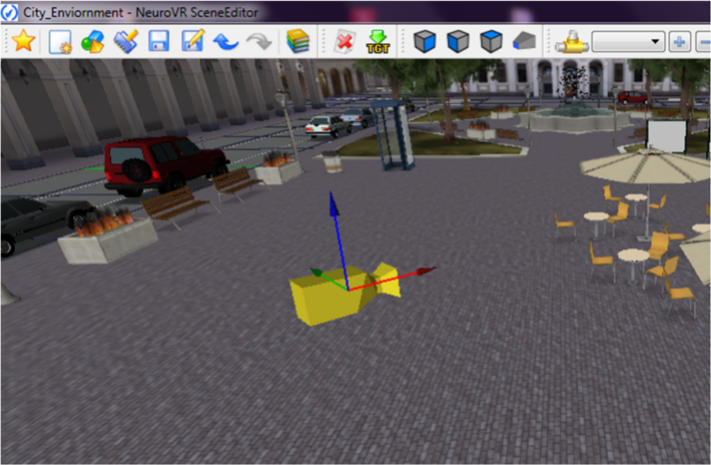
Camera position in the virtual environment. Researcher can also place a camera by pressing a button to set a new camera to reflect their vision in that precise moment

The immersive modality allows the scene to be visualized using a head-mounted display either in stereoscopic or in mono-mode. Compatibility with head-tracking sensor is also provided. In the non-immersive modality, the virtual environment can be displayed using a desktop monitor or a wall projector. The user can interact with the virtual environment using either keyboard commands, a mouse, or a joypad, depending on the hardware configuration chosen. In the editor, researcher can set the position of the camera to establish the first view of the users when the experiment begins (see Fig. 2).

It is possible to set more than one camera and to switch among different cameras using function keys. Moreover, it is possible to set the camera to follow the avatar movement or as a fixed camera. The download section of the platform’s website (www.neurovirtual.eu) provides the links to download the NeuroVirtual 3D installer and many contents free of charge. These programs include base contents pack (office, class, apartment, bivrs), body perception pack (scale, swimming pool, restaurant), green nature pack (lake, campfire, mountain, park, valley, waterfall), warm and sandy pack (beach, desert, gazebo, island, waves), shopping pack (supermarket and minimarket), public areas pack (auditorium, cinema, square), hospital and station.

**Fig. 3:**
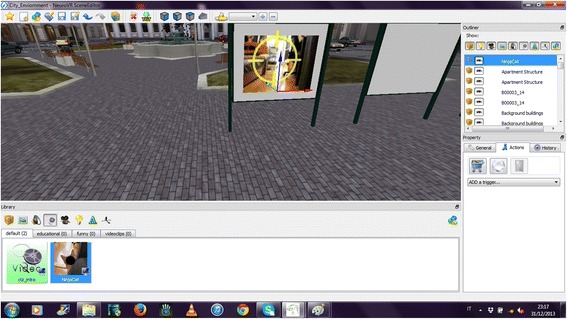
Drag-and-drop of a video, automatically fitted to the geometry of the environment

Many more 3D objects are available free of charge in the standard package. In the library, each 3D object as well as audio and video files, also the ones imported by the user (virtually in an unlimited number), are catalogued by categories. Object can be imported in the scene using a drag-and-drop function also using the perspective vision (such as in Fig. 3).

**Fig. 4:**
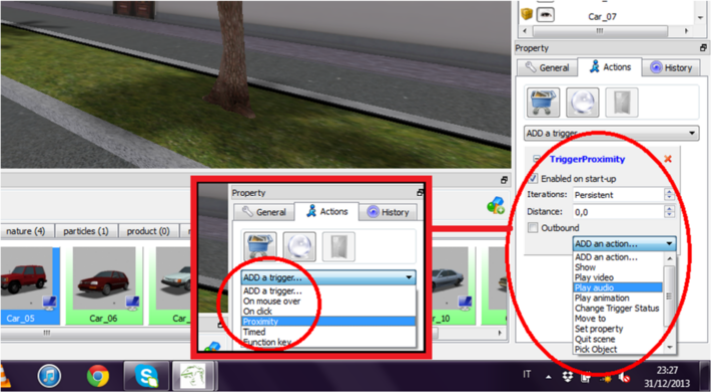
Trigger and action selection using simple menus. Once set, it is also possible to define specific parameters related to the trigger choice (for example number of interactions and distance when the trigger is set to “Proximity”)

Once an object is imported in the scene, it is possible to trigger it (Table 1) and to assign an action (Table 2), following the happening set with the specific trigger. For example, it is possible to play a sound when the user who is navigating the virtual environment is close to an object (e.g., a car). The trigger and action selection are set by the means of simple visual menu, as can be seen in Fig. 4.

**Table 1:** Triggering

Conditions	Satisfied when	Parameters
On mouse over	The mouse pointer is over the object	
On click	The object is clicked with the mouse	
On proximity	The camera is in proximity of the object	Activation distance, Inbound/Outbound
Timed	The *offset* time was elapsed, for a *Period* of time	Offset, Period
Function key	The specified function key was pressed on the keyboard	Function key from F1 to F12

**Table 2:** Action following the trigger

Actions	Description	Parameters
Show	Show or Hide the target object	Target object
Play video	Play selected video	Movie object, Type(Play, Pause, Stop), Loop(One time/Loop)
Play audio	Play audio	Sound object, Type(Play, Pause, Stop), Loop(One time/Loop)
Play animation	Play animation	Target object, Start time, Duration time, Loop(NumOf Times, Loop), Times
Change Trigger Status	Changed the Enable Status of the target object	Target object, Set Status To(Enabled, Disabled)
Move to	Move the object to the specified locator	Destination locator
Set property	Set a property of the target object to a new value	Target object, Property name, Property value
Quit scene	Quit the scene and close the NeuroVR Player	User message, System message
Pick object	The object is picked and placed in the tray bar	Target object to be picked
Load scene	Close current scene and open the new one without leaving the NeuroVR Player.	New scene to be loaded

**Fig. 5:**
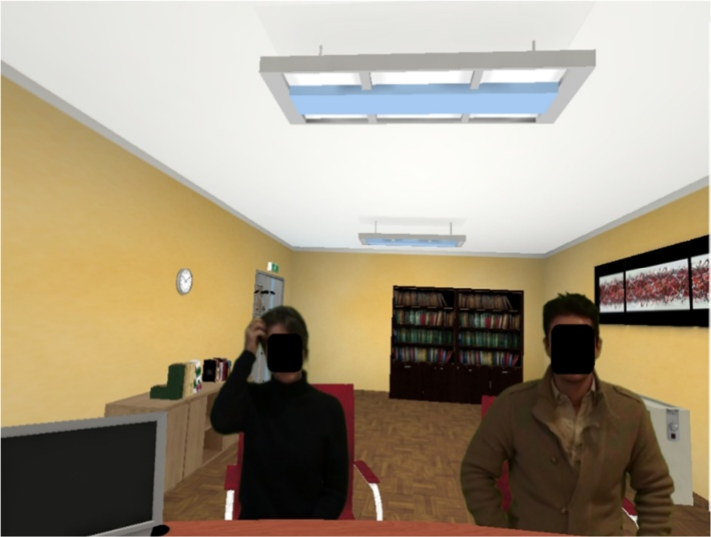
Real video in a virtual environment

It is also possible to create interacting video section based on pre-recorded narratives, which allows an effective virtual reality exposure using real video (with the alpha channel) within virtual environments (see Fig. 5) [11].

A making of a video production and importing objects in the scene can be seen through the following link made for a European project by a group of Italian researchers: http://youtu.be/vUlQ0JH6awI.

This capability to include video with alpha (transparence) channel made NeuroVirtual 3D a unique platform for research in behavioral science and clinical practice. In a very simple way, it is possible to run virtual reality exposure therapy (VRET), which would require very complex and expensive procedure alternatively. Increased capability to activate videos with a proximity trigger make virtual environment interactive and provide a vivid experience and immersion through an amplified sense of presence [9, 31].

Moreover, it is possible to create and import an avatar also by using the Autodesk Character Generator (https://charactergenerator.autodesk.com/) that is available online free of charge. These imported avatars can be rigged with several degree of freedom in the main body’s nodes, and finally, the avatar can also be configured through Virtual-Reality Peripheral Network (VRPN) using a Microsoft Kinect.

### Device and environment configuration

**Fig. 6:**
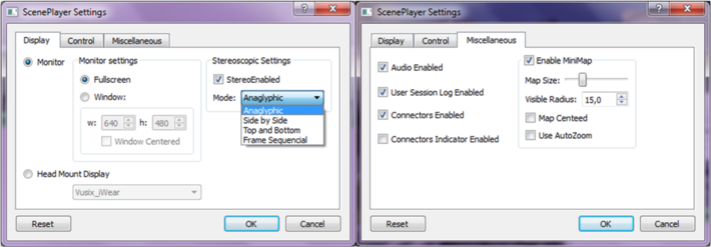
Display configurations and other settings

NeuroVirtual 3D provides a wide selection of visualizations both nonimmersive (through monitor or projectors) and immersive (through HMD) (Fig. 6). Nonimmersive navigation can be set on one or more monitors by adapting the desired resolution to fit the environment to the screens. Immersive navigation activates the tracker embedded in modern HMD, making the experiences fluid for the user who receives a visualization synchronized to his/her vestibular information. NeuroVirtual 3D can also embed a mini-map (size and radius can be defined; see Fig. 6), which is very important, as it provides allocentric information to the user during the navigation. The map has already been used in a study on spatial memory in elderly [27].

### Setting of input devices

Virtual environments have been traditionally used with a gamepad or keyboard and mouse. However, in the last few years, clinicians have began to be increasingly interested in new forms of interaction and arising clinical possibilities because of the proliferation of low cost devices. For example, the use of Kinect for motor neurorehabilitation has been widely tested [15]. Serious games have been considered as a possible approach with a higher ecological validity [33], however, as already mentioned, this approach lacks experimental flexibility. NeuroVirtual 3D platform can include Kinect in two different ways:

1. Navigation mode: user uses a set of gestures to navigate the environments, for example, rotating the shoulders 2° of freedom, s/he is able to move naturally in the environment (looking around) and with a step forward, s/he is also able to walk. An arm forward can be used as a mouse click.
2. Interaction mode: researcher can associate a gesture with a connector, which can be triggered in the editor. In this way, it is possible to interact with objects, videos, images, and anything else within the virtual environment by triggering elements to an action when a gesture is recognized. For example, a sound can be executed when the user moves an arm up.

**Fig. 7:**
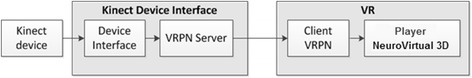
Input device can be set for navigation and interaction by using standard technologies

**Fig. 8:**
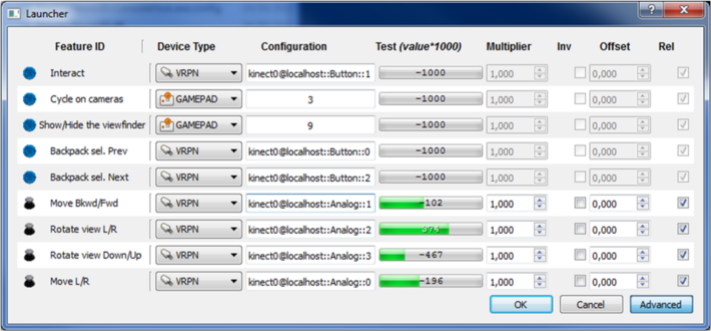
Input device can be set for navigation and interaction by using standard technologies, such as gamepads, keyboards, and mice and more advanced technologies, such as the Kinect or an Eye-tracker, by the means of VRPN protocol. The Launcher is also used to calibrate the device (here a Kinect), visualizing the thresholds (as can be seen in Test with green bars)

Navigation mode can be easily set through a graphic interface by selecting VRPN and defining the corresponding gesture (Fig. 7). Interaction mode is at the moment more complex, since it requires to open a config file (txt format) and changing parameters associated to each gesture (included angle degrees) manually. The configuration string in the Fig. 7 will pass the instruction to the device through VRPN. Within this framework, the user is able to calibrate the device for a perfect use (see green bars in Fig. 8).

### Biosensors and biofeedback

**Fig. 9:**
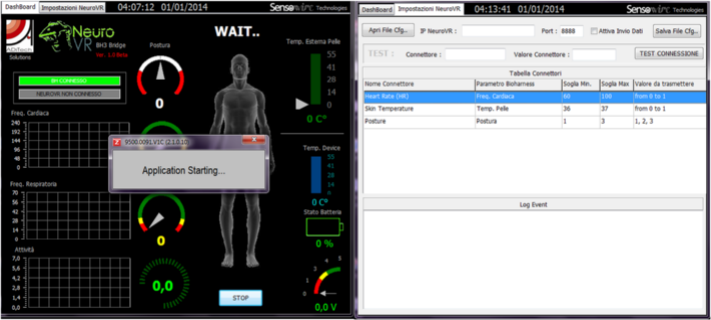
Configuration of Zephyr cardiac ECG (electrocardiography) strip biosensor to NeuroVirtual 3D to provide real time feedback

Psychophysiological measurement acquired an important role in experimental behavioral science, providing an effective psychometrics for specific measurements. The most important advantage of self-reported measures is that recording of these data is synchronous with the stimuli presentation. More importantly, in virtual environment, asking user to self-report the experience breaks the sense of presence conditioning the results. NeuroVirtual 3D is able to record psychophysiological data, synchronizing them with all events triggered into the environment (logging them) and thus providing an effective and simple way to collect this type of data in ecological settings. Moreover, NeuroVirtual 3D provides a direct feedback in real-time by triggering objects to specific indexes that can be defined according to the relevant literature, such as Malik for heart rate variability indexes [14]. In particular, NeuroVirtual 3D integrated a TCP-IP-based module (bridge) to collect the data from virtually any existent biosensor (Thought-Technology, Zephyr, and StarStim devices have already been included in the platform) (Fig. 9).

The feedback in virtual environments can be provided by adequately modifying specific objects or conditions. Five virtual environments are already provided in the biofeedback pack (beach, desert, lake, campfire, and park) where the environment light or specific objects (such as the fire in the campfire) change when physiological states change the established threshold values (see Fig. 8 as an example of physiological setting).

Indeed, the platform has been thought to stream the data in real time to obtain biofeedback as input from the biosensor, but following the same protocol (TCP/IP streaming of the physiological data), the platform can stream the data as output to any programs, including Matlab, which can be used to compute specific indices (like heart rate and heart rate variability indexes from electrocardiogram) and to classify the indexes using machine learning for affective computing [29]. The interface with computational models using NeuroVirtual 3D has already been tested [2].

### Logging

NeuroVirtual 3D is able to log any events in an ASCII file precisely within milliseconds (Unix timestamp) and identify specific events (trigger type, name and values). A complete log of all the coordinates and the direction vector is provided in Table 3.

**Table 3:** Event logging and description

Event keyword	Parameters	Description
CHANGE_SCENARIO	Scene unique	The first scenario is loaded or the current scenario is changed
QUIT, USER	-	The NeuroVR session is closed
PLAYER_MOVED	Transformation matrix: $$ \left(\begin{array}{c}\hfill {r}_x\hfill \\ {}\hfill {f}_x\hfill \\ {}\hfill {u}_x\hfill \\ {}\hfill {p}_x\hfill \end{array}\begin{array}{c}\hfill {r}_y\hfill \\ {}\hfill {f}_y\hfill \\ {}\hfill {u}_y\hfill \\ {}\hfill {p}_y\hfill \end{array}\begin{array}{c}\hfill {r}_z\hfill \\ {}\hfill {f}_z\hfill \\ {}\hfill {u}_z\hfill \\ {}\hfill {p}_z\hfill \end{array}\begin{array}{c}\hfill 0\hfill \\ {}\hfill 0\hfill \\ {}\hfill 0\hfill \\ {}\hfill 1\hfill \end{array}\right) $$	The last row contains the position of the user in the scene: $$ \left(\begin{array}{ccc}\hfill {p}_x\hfill & \hfill {p}_y\hfill & \hfill {p}_z\hfill \end{array}\right) $$ The second row contains the forward direction of the camera in the scene: $$ \left(\begin{array}{ccc}\hfill {f}_x\hfill & \hfill {f}_y\hfill & \hfill {f}_z\hfill \end{array}\right) $$
JOYPAD_KEYPRESSED	Button number	Joypad button pressed event
MOUSE_OVER	Object name	OnMouseOver trigger
CLICK	Object name	OnClick trigger event
PROXIMITY	Camera and object distance	Proximity Trigger event
TIMER_ELAPSED	Time in seconds	Time Trigger event
KEYBOARD_KEYPRESSED	Key code	Function key Trigger event

**Fig. 10:**
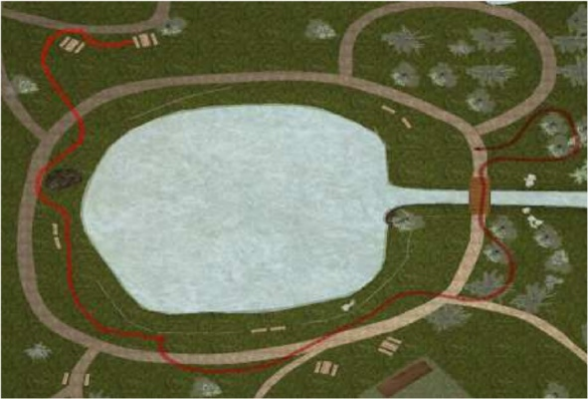
Automatically recorded map with the path generated by the user

Since all coordinates are available, the platform also automatically generates an environment mat with the path of navigation generated by the user (see Fig. 10).

### Limitations and future directions

NeuroVirtual 3D has some limitations that should be considered. First, although it does not require software programming skills, it is not practical for novice users. Clinicians without strong computer experience might have some difficulties in manipulating the objects, images, and videos to allow them to be seen from different angles (frontal, in perspective, and from the top), which is the standard procedure involved in identifying one’s correct position within the virtual environment. An initial training period is strongly suggested, as the tasks are generally complex and expectations of easiness need to be corrected. On the other hand, triggering procedures are very easy, and they can be performed by novices. Another limitation is the inaccessibility of devices that have not been tested. In fact, even if several devices have been tested and configured within the platform, researchers are continuously excited by new ones that could be not be tested yet and still need to be configured. Even if VRPN protocol is able to easily integrate several new devices, this procedure requires experience and computer expertise. Further, even more experience is needed if VRPN is not a standard of the producers; in that case, sdk integration or tcp/ip configuration will require some complex programming skills. In this sense, further efforts in future versions of the platform will need to include and test new devices and connect technical protocols to provide a wider range of connectable devices to the platform.

Clinicians have several reasons to shift their virtual reality experiments to a NeuroVirtual 3D platform, but the most crucial challenge will be to achieve wider use of VR and its full potential when combined with external devices and sensors. Indeed, VR facilitates experiments that would otherwise be very difficult or impossible in real environments. The power to move, scale, and rotate objects in psychological experiments (e.g., for perception or spatial memory) or to expose participants (also patients) to certain environments that would be impossible in the real world makes VR a powerful tool for experimental manipulation, exposure, and measurement in behavioral science. The big challenge of NeuroVirtual 3D is to make this environment easy and accessible to clinicians free of charge.

## Conclusion

Overall, it is clear that the main characteristics of the platform are easiness, accessibility, integration, and potential uses for clinicians. The experience acquired from previous versions of the platform facilitated testing in experimental and clinical settings on patients with different conditions, in particular Stroke [18], obsessive compulsive disorders [4, 13], generalized anxiety disorders [16, 19, 20], Parkinson’s [3], Neglect [17], Eating Disorders [28], and Schizophrenia [13], among others. Additionally, VR has been used in exposure therapy for fear, stress, and anxiety and in assessment protocols. Studies on the use of VR for motor and neuro rehabilitation have provided new clues for further development as well as a more accurate and validated tool for clinicians.

At the moment, NeuroVirtual 3D represents the most advanced free technology for experimental behavioral science using virtual environments currently available without the need to program the codes. Compared to other platforms, NeuroVirtual 3D is impressive primarily for being available free of charge to everyone as well as for its main characteristics that make this platform the most advanced at the moment (see Appendix for a comparison). In particular, the integration of VRPN module allows virtually any device to be connected easily, including biosensors for biofeedback and affective computing. Furthermore, the drag and drop design makes the platform very easy to use, and the ability to integrate multiple media formats (3D objects, pictures, videos, sounds, etc.) enriches its contents. Additionally, the triggering system makes it very interactive, and the logging system makes it perfect for research and precise millisecond experiments. Finally, the availability of validation protocols and several use of its precursors make this platform a first-class cutting-edge technology for clinical and experimental sciences.

Researchers and clinicians are now able to work in their lab running ecological experiments and clinical protocols in patients with reduced motility.

## Availability and requirements

**• Project name:** NeuroVirtual 3D

**• Project home page:** http://www.neurovirtual.eu

**• Operating system(s):** Window Platform (32bit and 64 bit platforms)

**• Programming language:** No programming language is required for using the software. Regarding VRPN configuration, the client interfaces are written in C++ but has been wrapped in Python and Java.

**• Other requirements:** Windows XP or higher

**• License:** Available for free

**• Any restrictions to use by non-academics:** No restrictions

## Appendix

Following a benchmarking table of existent alternative platorms (Table 4).

**Table 4:** A benchmarking table of existent alternative platforms

	Platform name			
	Virtually better	Virtual ret	Virtual reality rehabilitation system	NeuroVirtual3D
Owner and license	Virtually Better (Proprietary Software)	Virtual Ware (Proprietary Software)	Khymeia (Proprietary Software)	Istituto Auxologico Italiano (FREE Software)
Website	www.virtuallybetter.com	www.virtualret.es/es/inicio/1.html	www.khymeia.com	www.neurovirtual.eu
Primary users target	Psychotherapy (PTSD)	Psychotherapy (anxiety disorders)	Motor neurorehabilitation	Experimental psychology, Psychotherapy and Neurorehabilitation
Market of interests	International	Mostly Spanish	Mostly Italian	International
Report	Log user activity	Log user activity	Log user activity, performance and functional recovery measure	Log user activity, performance and functional recovery measure, territorial map, trigger logging and psychophysiological measures
hardware third parts support	NO	NO	NO	YES
Available SDK	NO	NO	NO	YES
Multi-user support	YES	YES	NO	YES
Online sharing of virtual environments and scenarios	NO	NO	NO	YES
Experimental validation protocols	YES	Data non available	YES	YES
Customizing virtual environments treatment	YES	NO	It can create new exercises using the built-in library of 3D objects	Users can create new exercises using the multimedia editor included with the software
Integration of multimedia content in 3D environments	YES	NO	NO	YES, users can use the editor to insert audio and video clips in virtual environments
Portable exercises mode	NO	NO	YES	Yes, some environments will be available on the mobile platform (Android or iPhone) in the future
Eye-tracking Integration	NO	NO	NO	YES
telecare / remote assistance mode	YES	NO	YES	Yes, a therapist may use remote environments with the patient
Possibility of customization / personalization software	YES	Data not available	YES	YES
Further hardware integration (biosensors, devices, etc.)	NO	NO	NO	YES, though VRPN or TCP/IP protocol (dedicated port)

## Abbreviations

HMD: Head-mounted display
VR: Virtual reality
Sdk: Software developer kit
VEs: Virtual environments
VRET: Virtual reality exposure therapy
VRPN: Virtual-reality peripheral network
